# Implementation of Function-Focused Care in the Hospital Setting: Results of the FFC-AC-EIT Trial

**DOI:** 10.1093/geroni/igaf122.721

**Published:** 2025-12-31

**Authors:** Barbara Resnick, Marie Boltz

## Abstract

Once hospitalized, individuals living with dementia are more likely to experience negative impacts of hospitalization including a decline in physical activity, function, falls, delirium, and behavioral symptoms associated with dementia, when compared to individuals without cognitive impairment. The Function Focused Care for Acute Care Using the Evidence Integration Triangle (FFC-AC-EIT) intervention, in contrast to structured exercise or mobility programs, helps nurses engage patients in routine care activities and therefore provides a more realistic and sustainable approach to increase function and physical activity. The FFC-AC-EIT intervention was implemented by a nurse interventionist working with a unit-based interdisciplinary stakeholder team and champion using a four step approach: environment and policy assessment; education of staff, patients, and families; development of function-focused care goals; and mentoring and motivating the staff, patients, and families. A clustered randomized design was used with 12 hospitals randomized to FFC-AC-EIT versus Education Only. This symposium reports the main outcomes of the FFC-AC-EIT trial, as well as other relevant, secondary analyses that provide insights into factors to consider when implementing and evaluating FFC-AC-EIT. The first and second presenters will report the primary and secondary outcomes of the trial, respectively. The third presenter will discuss the impact of delirium upon our major outcome, physical function, in a sample of older adults with dementia. Finally, the fourth presenter will describe the association between patient engagement and the quality of staff-patient interactions.

